# Pilot study on the distribution of caseous lymphadenitis in Korean native goats and the relationship between sex and age in disease occurrence

**DOI:** 10.3389/fvets.2023.1274359

**Published:** 2023-11-28

**Authors:** Md. Aftabuzzaman, Jaylord M. Pioquinto, Hector Espiritu, Edeneil Jerome Valete, Seon-Ho Kim, Su-Jeong Jin, Myunghwan Jung, Sang-Suk Lee, Yong-Il Cho

**Affiliations:** ^1^Department of Animal Science and Technology, College of Bio-Industry Science, Sunchon National University, Suncheon, Republic of Korea; ^2^Department of Microbiology, College of Medicine, Gyeongsang National University, Jinju, Republic of Korea

**Keywords:** caseous lymphadenitis, Korean native goats, distribution, sex, age

## Abstract

Caseous lymphadenitis (CLA) caused by *Corynebacterium pseudotuberculosis* is a chronic disease that affects goats. The Korean native goat (KNG) is the most popular goat breed raised in Korea. In this study, the distribution of CLA in the KNG population and the relevance of sex and age in disease development were determined. From March 2020 to February 2021, 1,177 KNGs from 110 farms were assessed. The distribution of CLA in animals was determined by a veterinary inspector who performed gross examinations of goat carcasses and confirmed diagnoses via polymerase chain reaction and bacteriological examination. The CLA detection rate in the KNG population was 19.80%, and more than half of the farms were affected by the disease (56.36%). A statistically significant difference was observed among the sex groups, with castrated males (13.98%) having the lowest detection rate, followed by intact males (22.48%) and females (24.09%), suggesting that castration has a positive effect on reducing the risk of CLA (*p* < 0.05). In terms of age groups, an increased detection rate of 28.16% was observed in the late adult (>2 years) group. Phylogenetic analysis indicated that the RNA polymerase beta subunit-encoding gene can effectively differentiate biovar *ovis* from biovar *equi* and can be used for further epidemiological studies of *C. pseudotuberculosis*. This is the first nationwide surveillance study of CLA distribution to confirm the continuous occurrence of CLA in Korean goat farms. Future studies should include risk factor analyses of CLA based on herd levels to prevent and control this disease in Korea.

## 1 Introduction

Caseous lymphadenitis (CLA) is a chronic, recurrent, and highly contagious infection caused by *Corynebacterium pseudotuberculosis*, a gram-positive, pleomorphic, facultative, anaerobic intracellular bacterium (1) that can infect a wide range of animal hosts, including humans, and is considered a potential zoonotic disease (2, 3). Individuals in direct contact with infected animals, such as abattoir workers, farmers, and veterinarians, are at high risk of contracting the disease (4). Ten cases of human lymphadenitis have been documented in Australia, as well as a case of *C. pseudotuberculosis* pneumonia in a veterinary student who was infected during laboratory work (4, 5). Clinical signs in humans include lethargy, malaise, inflammation, and abscessation of one or more lymph nodes (4).

*C. pseudotuberculosis* must penetrate the skin or mucous membranes to establish an infection (2). The main transmission modes are either direct contact with the pus of abscesses from the affected animal during close confinement, or indirect contact through the sharing of contaminated devices used in ear tagging and other husbandry practices (1, 3). The main source of transmission in infected animals is a granulated fistula or ruptured abscess (6). Infected animals may exhibit lesions characterized by purulent encapsulated abscesses with a caseous onion ring appearance (7). Furthermore, animals with CLA may develop external lesions characterized by enlarged skin and peripheral lymph nodes, resulting in carcass condemnation during slaughter (8). Internal lesions are characterized by the development of granulomatous lymphadenitis with abscessation (6, 9). External lesions primarily affect the superficial lymph nodes, whereas internal lesions affect the lungs, liver, kidneys, and heart (1, 6). The presence of an external abscess in the peripheral lymph nodes of small ruminants is highly suggestive of CLA (1); however, a definitive diagnosis can only be made based on a bacterial culture of purulent material from an intact abscess (10). This differentiates it from other pyogenic organisms, such as *Trueperella pyogenes*, *Staphylococcus aureus*, *Pasteurella multocida*, and *Actinobacillus licheniformis*, which can also cause abscessation (10, 11). Direct and indirect tests to detect *C. pseudotuberculosis* have already been proposed, such as the complement fixation test, synergistic hemolysis inhibition test, microagglutination assay, phospholipase D (PLD) antigen-based enzyme-linked immunosorbent assay (12), and polymerase chain reaction (PCR) targeting the 16S rRNA, PLD, and RNA polymerase beta subunit-encoding gene (*rpoB*) genes for the differentiation of CLA at the biovar level (13).

CLA is a major concern in the small ruminant industry because of its global distribution and the difficulty of disease control and mitigation. This has resulted in significant economic and production losses (14). CLA can infect goats of any breed, age, or sex; however, these host-related factors can affect the distribution and prevalence of the disease (15, 16).

The Korean native goat (*Capra hircus coreanae*) (KNG) is a goat breed indigenous to Korea (17), and is the only goat species officially registered by the Food and Agriculture Organization in Korea (18, 19). According to the Ministry of Agriculture and Forestry, the goat population has increased two-fold, from approximately 242,000 in 2010 to 443,000 in 2021. This increase in the domestic goat industry is mainly attributed to consumer demand and preference for goat meat as a healthy food (17, 19). The growth of goat populations has attracted the interest of farm producers regarding disease control, feeding management, and goat breeding, as well as veterinary personnel.

Currently, little information is available regarding CLA levels in KNGs in Korea. This scarcity of data has led to a limited understanding of the epidemiology, pathogenesis, and disease management of CLA in KNGs. Thus, the aims of this pilot study were to investigate the distribution of CLA in KNGs, evaluate the association between sex, age, and disease occurrence, and generate molecular biological data for CLA in Korea. This information can be utilized in Korean goat husbandry and may contribute to the implementation of CLA prevention and control measures on farms.

## 2 Materials and methods

### 2.1 Ethics approval statement

All experimental protocols were approved by the Animal Care and Use Committee (approval number: SCNU IACUC-2020-3) of Sunchon National University (Suncheon, Korea). All experiments were performed in accordance with the guidelines and regulations set by the governing body.

### 2.2 CLA surveillance and categorization

The presence of CLA in KNGs was assessed at a goat abattoir in Jeonnam Province, Korea. CLA lesions were collected twice a month. A total of 1,177 KNGs from 110 goat farms were evaluated between March 2020 and February 2021. A veterinary inspector examined all carcasses to detect CLA lesions. CLA lesions were identified as external lesions based on their regional location in the front/proximal, middle, or hind/distal parts of the body. The lesions were characterized by enlargement of the skin and peripheral lymph nodes. All carcasses were examined by a veterinary inspector to detect CLA lesions. The collected CLA lesions were transported to the laboratory for further analysis in an insulated cooler box maintained at a temperature of 4°C. A farm with at least one animal showing signs of CLA lesions was considered CLA positive. Animals with CLA lesions were classified into three sex groups: female, intact male, and castrated male; and three age groups: young (< 1 year), adult (1–2 years), and late adult (> 2 years).

### 2.3 Molecular detection

For the PCR detection of *C. pseudotuberculosis*, DNA was extracted from pus samples and isolates using a QIAamp DNA Mini Kit (Qiagen, Germany) according to the manufacturer’s instructions. The DNA was stored at −20°C until further use. The detection of suspected *C. pseudotuberculosis* was confirmed via PCR amplification of the PLD gene (Table 1) according to previous studies (20, 21), with slight modifications. The cycling conditions were 98°C for 2 min of initial denaturation, 30 cycles of denaturation at 98°C for 20 s, annealing at 56°C for 20 s, extension at 72°C for 20 s, and a final extension at 72°C for 3 min. The PCR products were electrophoresed on a 1.5% (w/v) agarose gel using GreenStar nucleic acid staining solution (Bioneer, Republic of Korea) and visualized under ultraviolet light.

**Table 1:** The oligonucleotide primers used for PCR assay.

Target gene	Primers	Sequence (5′ – > 3′)	Product size (bp)
PLD	PLD-F	ATAAGCGTAAGCAGGGAGCA	203
	PLD-R2	ATCAGCGGTGATTGTCTTCCAGG	
*rpoB*	C2700-F	CGTATGAACATCGGCCAGGT	446
	C3130-R	TCCATTTCGCCGAAGCGCTG	
16S rRNA	27F	AGAGTTTGATCMTGGCTCAG	1,500
	1492R	TACGGYTACCTTGTTACGACTT	

### 2.4 rpoB gene and 16S rRNA amplification and sequencing

The *rpoB* and 16S rRNA were amplified using the following cycling conditions: 3 min of initial denaturation at 94°C, 35 cycles at 94°C for 1 min, at 56°C for 1 min, 72°C for 2 min, and a final elongation of 7 min at 72°C. PCR products were sequenced in both forward and reverse directions via Sanger sequencing, which involves the selective incorporation of chain-terminating dideoxynucleotides by DNA polymerase during *in vitro* DNA replication, by Macrogen, Inc. (Seoul, Korea).

### 2.5 Phylogenetic analysis

The *rpoB* gene and 16S rRNA sequences of *C. pseudotuberculosis* from this study and those of different species, including goats, sheep, horses, alpacas, and camels, were retrieved from the NCBI database^1^ and used for phylogenetic analyses. The Basic Local Alignment Search Tool was used to compare the detected sequences with existing sequences in the GenBank database. The partial sequences were aligned using the ClustalW algorithm in the Molecular Evolutionary Genetic Analysis (Mega v.11) software. A phylogenetic tree was constructed using a maximum likelihood test with a bootstrap value of 1,000 repetitions and reference sequences from different species in GenBank.

### 2.6 Statistical analysis

The total number of animals examined, as well as, sex, age, and number of animals with CLA lesions observed during the gross examination were recorded. Individual and farm-level distributions were calculated as percentages. Frequency distributions and percentages were used to analyze the occurrence of CLA in terms of sex and age. Statistical analysis was performed by descriptive statistics using crosstabs, and the difference was estimated using Pearson’s chi-square test and SPSS 21.0. *p*-values lower than 5% (*p* < 0.05) were considered significant.

## 3 Results

### 3.1 Distribution of CLA in KNGs

The distribution of CLA at an individual level was 19.80%. More than half of the goat farms (56.36%) where KNGs were slaughtered had animals with CLA lesions. The distribution of CLA at both the individual and farm levels among the KNGs examined is shown in Table 2.

**Table 2:** Distribution of CLA among individual Korean native goats and farm level.

Categories		Number	CLA positive	Distribution (%)
Individual	(Total goats)	1,177	233	19.80
Farm	(With at least 1 case)	110	62	56.36

### 3.2 Distribution of CLA in relation to sex and age

The distribution of CLA lesions in KNGs differed among the sex groups. Castrated males had the lowest recorded occurrence of CLA, with 65 out of 465 animals identified as having CLA lesions, representing a detection rate of 13.98%. This was followed by intact males, in which 49 out of 218 animals were affected by CLA lesions, representing a detection rate of 22.48%. Females had the highest incidence of CLA (24.09%, 119/494). CLA was distributed significantly less frequently in castrated males than in females or intact males; however, no significant difference was observed between females and intact males (Table 3).

**Table 3:** Distribution of CLA in Korean native goats based on sex and age.

Variable	Category	Number of animals examined	Number of CLA positive animals (%)	Chi-square (χ2)	*p*-value
Sex	Female	494	119 (24.09^b^)	16.633	0.01
	Intact male	218	49 (22.48^b^)		
	Castrated male	465	65 (13.98^a^)		
Age	Young (under one year)	288	50 (17.36)	5.629	0.06
	Adult (one to two years)	786	154 (19.59)		
	Late adult (over two years)	103	29 (28.16)		

Different letters (a, b) indicate significant statistical difference (*p* < 0.01).

Of the 1,177 animals examined for the presence of CLA lesions, 103 were > 2 years old (late adults), 786 were between 1 and 2 years old (adults), and 288 were < 1 year old (young). Of these animals, 29 late adults, 154 adults, and 50 young KNGs were grossly affected by CLA, with detection rates of 28.16, 19.59, and 17.36%, respectively (Table 3). No significant differences were observed among the age groups. However, the occurrence of CLA tended to increase as the age of the KNGs increased (*p* = 0.06).

### 3.3 Distribution of study population based on sex and age groups

A total of 786 goats were examined in the adult group, with 288 and 103 goats in the young and late adult groups, respectively. The adult group consisted mainly of females, followed by castrated males, with 391 and 374 animals, respectively. Intact males (197 animals) dominated the young age group, whereas 100 female KNGs contributed to the highest number examined in the late adult age group. The distribution of animals was dependent on the number of animals slaughtered during the study period (Table 4).

**Table 4:** Distribution of study population and number of goats with caseous lymphadenitis.

Age	Sex			Total
	Number of castrated males/positive animals	Number of intact males/positive animals	Number of females/positive animals	
Young	88/8	197/40	3/2	288/50
Adult	374/54	21/9	391/91	786/154
Late adult	3/3	0	100/26	103/29
Total	465/65	218/49	494/119	1177/233

### 3.4 Phylogenetic analysis of *Corynebacterium pseudotuberculosis*

**Figure 1:**
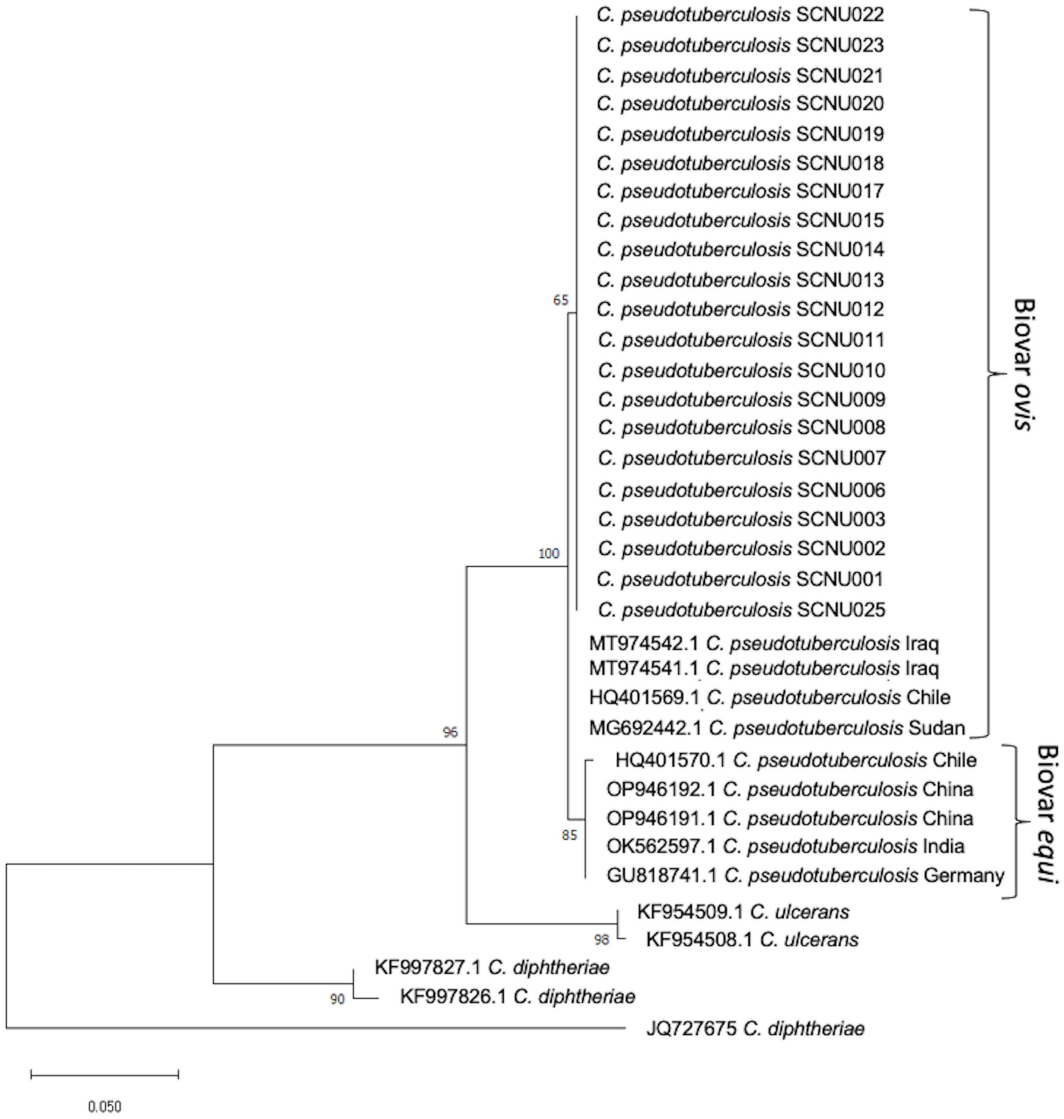
The phylogenetic tree based on the sequence of *rpoB* gene was constructed by MEGA11 software using Maximum Likelihood method based on the Tamura 3-parameter model with a bootstrap value of 1,000 repetition. The bar represents 0.050 substitutions per nucleotide position. 35 *C. pseudotuberculosis* isolates (21 from this study and 14 from GenBank reference samples), a reference sequences of the *C. ulcerans*, *Corynebacterium renale* and *Corynebacterium diphtheria* were used in the analysis.

**Figure 2:**
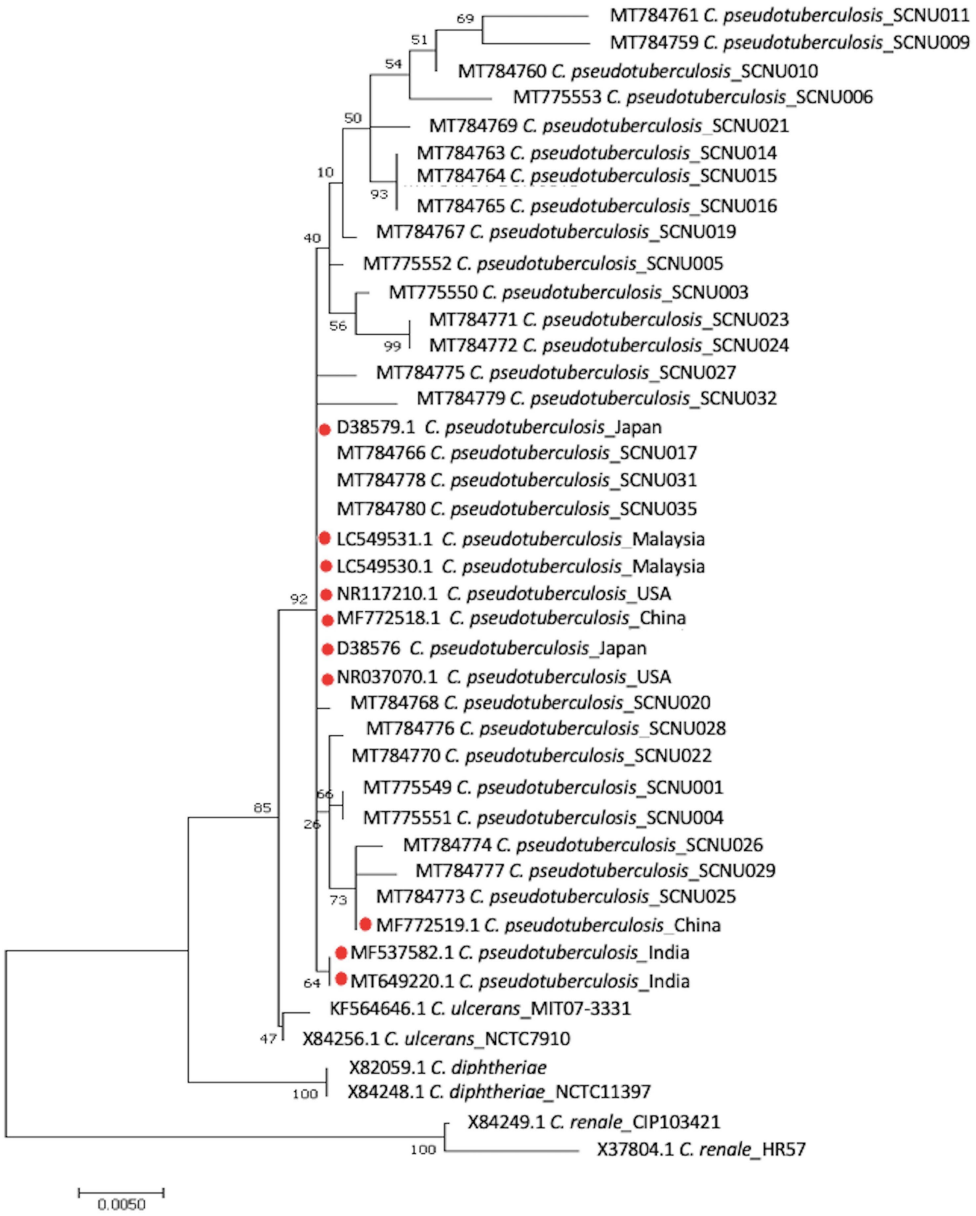
The phylogenetic tree based on the sequence of 16S rRNA gene was constructed by MEGA11 software using Maximum Likelihood method based on the Hasegawa-Kishino-Yano model with a bootstrap value of 1,000 repetition. The bar represents 0.005 substitutions per nucleotide position. 36 *C. pseudotuberculosis* isolates (26 from this study and 10 from GenBank reference samples), a reference sequences of the *C. ulcerans*, *C. renale* and *C. diphtheria* were used in the analysis.

Twenty-one *rpoB* gene and 26 16S rRNA sequences were obtained from *C. pseudotuberculosis* after amplification and sequencing. The percentage of similarity of the 16S rRNA and *rpoB* gene partial sequences of our isolates ranged between 98% and 100% with respect to sequences reported globally. The phylogenetic tree based on *rpoB* gene sequences showed that all of our isolates were grouped with *C. pseudotuberculosis* biovar *ovis* and could be clearly differentiated from biovar *equi* as they formed two separate clades (Figure 1). The use of 16S rRNA gene sequences facilitated easy differentiation of *C. pseudotuberculosis* from other *Corynebacterium* spp., but was ineffective for differentiating biovar *ovis* from biovar *equi* (Figure 2). Phylogenetic analysis revealed that all isolates were clustered in one group together with reference isolates from different countries, such as Iraq, Chile, Sudan, China, India, and Germany, for the *rpoB* gene, and Japan, Malaysia, India, USA, and China for 16S rRNA. *Corynebacterium ulcerans* was found to be the species closest to *C. pseudotuberculosis*.

## 4 Discussion

In this study, gross examination, bacteriological investigation, and molecular detection revealed that CLA is endemic to KNG. Among the 1,177 examined animals, 233 exhibited typical CLA lesions, representing an individual detection rate of 19.80%. The distribution of CLA at the farm level was 56.36%, suggesting that more than half of the farms had at least one CLA-positive goat. Epidemiological studies in other countries that performed clinical examinations to identify CLA prevalence included studies in Brazil (25.33%) (22), Egypt (11.04%) (15), two separate studies in Ethiopia (18.80 and 15.00%) (8, 16), and India (4.38%) (23). The differences in CLA prevalence between different studies may be attributed to the variation in management systems and climatic conditions in each study, as the viability of the causative organism in the contaminated environment is greatly affected by ambient temperature. They may also be attributed to the endemic nature of the disease, which leads to variations in animal immunity and degree of animal susceptibility (15). Furthermore, variations in the use of different diagnostic tools for determining CLA may contribute to differences in CLA prevalence among different countries (8, 16, 22). In the present study, CLA lesions were detected through direct examination of the carcass, and the etiology of the lesions was confirmed using PCR. To date, this is the only study on the distribution of CLA in Korea that focuses on the direct examination of animals by a veterinary inspector as a method for identifying CLA lesions. In contrast, other studies in Korea only focused on serological prevalence, in which 57.30% (24) and 43.00% (25) of animals were seropositive. These results suggest that the disease is widespread in Korea. Although CLA is widely prevalent in goats, studies of CLA in Korea are lacking. This study was conducted over one year and included a regular survey of a goat slaughterhouse. Information regarding the current distribution of CLA, including its specific status in Korea, will be beneficial for establishing appropriate preventive measures.

The data gathered from this study allowed for the evaluation of CLA distribution with respect to the sex of the animals. To the best of our knowledge, this is the first study to classify male goats as castrated or intact. The distribution of CLA was found to be significantly lower in castrated male KNGs than in females and intact males (*p* < 0.05). Castration reduces aggressive behavioral problems by eliminating testicular androgens, implying that a lack of aggression results in fewer combat injuries that could serve as an entry route for the pathogen, a factor underlying the lower incidence of CLA in castrated males (26). Most castrated males were reared until they were one to two years of age, similar to females, to increase animal size before culling. The distribution of CLA in female KNGs was considerably high compared to that in others. This may be because most females were older, and a longer duration of stay might make them more susceptible to CLA, which explains the higher distribution (22). Our data showed that the female population was large during the adult and late adult stages, and small at young ages. Moreover, those remaining on farms for more than two years are susceptible to chronic CLA development. Adult CLA-positive animals are a potential risk factor for infection because they maintain the pathogen on farms and among herds (27). Another reason for the higher CLA levels in this group is that females can be reared together as they are less aggressive than males (28). This greater level of contact among females may result in a higher CLA occurrence (16). Additionally, disorders during pregnancy, such as pregnancy toxemia, which results in immunosuppression by inhibiting leukocyte function, may increase the progression of diseases such as CLA in female goats (29).

In terms of age predisposition for CLA distribution, this study showed that CLA occurrence tended to increase in the late adult KNG population. The greater prevalence of CLA in the late adulthood group may be explained by the chronic nature of the disease (27). An animal that stays on a farm longer has an increased probability of coming into contact with the pathogen, and can infect and reinfect other animals in the herd (22). This implies that older animals that stay longer in the herd increase the chances of exposure to the pathogen through numerous risk factors, such as herd size, housing, farm hygiene, and herd and health management for CLA infection (30). This result is consistent with those of previous studies on different goat breeds, where adult and late-adult animals had a significantly higher prevalence of CLA (8, 22). However, other studies have contradicted this data, reporting that age was not associated with seropositivity or bacterial identification in clinical cases of CLA (30). This is supported by a similar study showing that age had no significant effect on CLA infection in a goat herd. Multivariate factors can affect disease development and alter the outcomes of different studies (31). Consequently, the association between animal age and CLA occurrence is affected by many variables, such as differences in feeding, management, or environment (30). Most of the animals in each group were late adults and adult females, followed by young intact males. These differences in the number of animals in different age groups are due to market requirements and consumer preferences, particularly when meat quality without the characteristic odor is considered important (22). The same reason accounted for the decrease in the number of late-adult KNGs, especially intact males; no late-adult intact males were examined. Most female KNGs were in the late-adult age group, primarily because they are reared longer for breeding and milking purposes.

Molecular methods, including restriction fragment length polymorphism of chromosomal DNA, ribotyping, and whole genome sequence analysis, have been used to differentiate *C. pseudotuberculosis* biotypes (32–34). Recently, two biotypes of *C. pseudotuberculosis* were differentiated using concatenated partial sequences of four housekeeping genes (*dnaK*, *groEL1*, *infB*, and *leuA*) (35). In this study, we used the *rpoB* gene and 16S rRNA to classify the relationships between different isolates. Although the 16S rRNA gene sequence has been the most frequently used molecular marker for bacterial identification and phylogenetic analysis, analysis of *C. pseudotuberculosis* at the biovar level has shown that the internal region of the RNA polymerase beta subunit-encoding gene (*rpoB*) is a more suitable sequence than 16S rRNA (36, 37) due to the high nucleotide polymorphism in the *rpoB* gene (36, 37). In this study, we found that the *rpoB* gene can effectively differentiate biovar *ovis* from biovar *equi* of *C. pseudotuberculosis* and can be used for further epidemiological studies of this pathogen. Similar research confirmed that phylogenetic analysis based on the *rpoB* gene can confirm subspecies differentiation (38).

This study on CLA distribution in KNGs suggests that CLA is endemic to Korea; however, the gathered epidemiological data may not fully reflect the actual situation in Korea because of the limited number of slaughtered animals examined. Therefore, future studies should include larger animal populations and more farms to accurately estimate the prevalence of CLA in KNGs. Furthermore, an equal distribution of animals in both age and sex groups may reveal additional factors that could aid in understanding disease occurrence and infection patterns within the KNG population.

## 5 Conclusion

In this study, the distribution of CLA in KNGs at the individual (19.80%) and farm (56.36%) levels was investigated. The present study is the first to provide insights into how age and sex management can predispose animals to CLA by categorizing male KNGs as either intact or castrated. Although castrating animals may not fully eliminate the risk of CLA infection, this study demonstrated that it can significantly reduce the likelihood of contracting CLA infection. Older and female subjects were more strongly affected than any other age or sex group. In the future, risk factor analysis of CLA based on the current findings may help to prevent and control this disease in Korea. A phylogenetic tree of partial sequences of the *rpoB* gene showed that biovar *ovis* isolates from both sheep and goats were grouped together in the same lineage, confirming that the *rpoB* gene allows differentiation of *C. pseudotuberculosis* isolates at the biotype level.

## Data availability statement

The original contributions presented in the study are publicly available. This data can be found at: https://www.ncbi.nlm.nih.gov/nuccore; OR361299 – OR361319.

## Ethics statement

The animal studies were approved by Animal Care and Use Committee (approval number: SCNU IACUC-2020-3) of the Sunchon National University (Suncheon, Korea). The studies were conducted in accordance with the local legislation and institutional requirements. Written informed consent was not obtained from the owners for the participation of their animals in this study because animals used are the animals sent to slaughterhouses.

## Author contributions

MA: Data curation, Investigation, Writing – original draft, Writing – review & editing. JP: Writing – original draft, Writing – review & editing. HE: Investigation, Supervision, Writing – review & editing. EV: Writing – review & editing. S-HK: Data curation, Writing – review & editing. S-JJ: Data curation, Writing – review & editing. MJ: Supervision, Visualization, Writing – review & editing. S-SL: Supervision, Visualization, Writing – review & editing. Y-IC: Conceptualization, Funding acquisition, Project administration, Resources, Supervision, Visualization, Writing – review & editing.

## Footnotes

^1^ http://www.ncbi.nlm.nih.gov/genbank/

## References

[1] Dorella FA Pacheco LGC Oliveira SC Miyoshi A Azevedo V. *Corynebacterium pseudotuberculosis*: microbiology, biochemical properties, pathogenesis and molecular studies of virulence. Vet Res. (2006) 37:201–18. doi: 10.1051/vetres:2005056 16472520

[2] Williamson LH. Caseous lymphadenitis in small ruminants. Vet Clin North Am Food Anim Pract. (2001) 17:359–71. doi: 10.1016/S0749-0720(15)30033-5 11515406

[3] Guimarães AS Carmo FB Pauletti RB Seyffert N Ribeiro D Lage AP. Caseous lymphadenitis: epidemiology, diagnosis, and control. IIOAB J. (2011) 2:33–43.

[4] Peel MM Palmer GG Stacpoole AM Kerr TG. Human lymphadenitis due to *Corynebacterium pseudotuberculosis*: report of ten cases from Australia and review. Clin Infect Dis. (1997) 24:185–91. doi: 10.1093/clinids/24.2.185, PMID: 9114145

[5] Heggelund L Gaustad P Håvelsrud OE Blom J Borgen L Sundset A. *Corynebacterium pseudotuberculosis* pneumonia in a veterinary student infected during laboratory work. Open Forum Infect Dis. Oxford University Press. (2015) 2:ofv053. doi: 10.1093/ofid/ofv053 26380345 PMC4567093

[6] Fontaine MC Baird GJ. Caseous lymphadenitis. Small Rumin Res. (2008) 76:42–8. doi: 10.1016/j.smallrumres.2007.12.025

[7] Domenis L Spedicato R Pepe E Orusa R Robetto S. Caseous lymphadenitis caused by *Corynebacterium pseudotuberculosis* in alpine chamois (Rupicapra r. rupicapra): a review of 98 cases. J Comp Pathol. (2018) 161:11–9. doi: 10.1016/J.JCPA.2018.04.003, PMID: 30173853

[8] Zeru F Kahsay AG. Caseous lymphadenitis in goats from Borena range land South Ethiopia slaughtered at Luna export abattoir. J Vet Med Anim Heal. (2014) 6:168–73. doi: 10.5897/JVMAH2013.0251

[9] Oreiby AF. Diagnosis of caseous lymphadenitis in sheep and goat. Small Rumin Res. (2015) 123:160–6. doi: 10.1016/j.smallrumres.2014.11.013

[10] Shin D-H Song Y-K Byun J-W Kim H-Y Kim H-S Woo G-H. Caseous lymphadenitis by *Corynebacterium pseudotuberculosis* in a Saanen dairy goat (*Capra hircus aegagrus*). Korean J Vet Res. (2010) 50:25–8.

[11] Pekelder JJ. Caseous lymphadenitis. Martin W.B. Aitken I.D. Diseases of sheep. 3rd edn, Blackwell Science, Oxford (2003)

[12] Dercksen DP Brinkhof JMA Dekker-Nooren T van Maanen K Bode CF Baird G. A comparison of four serological tests for the diagnosis of caseous lymphadenitis in sheep and goats. Vet Microbiol. (2000) 75:167–75. doi: 10.1016/S0378-1135(00)00217-0, PMID: 10889407

[13] Gonçalves JL Tomazi T Barreiro JR Braga PAC Ferreira CR Araújo Junior JP. Identification of Corynebacterium spp. isolated from bovine intramammary infections by matrix-assisted laser desorption ionization-time of flight mass spectrometry. Vet Microbiol. (2014) 173:147–51. doi: 10.1016/j.vetmic.2014.06.028, PMID: 25086477

[14] Baird GJ Fontaine MC. Corynebacterium pseudotuberculosis and its role in ovine Caseous lymphadenitis. J Comp Pathol. (2007) 137:179–210. doi: 10.1016/J.JCPA.2007.07.002 17826790

[15] Al-Gaabary MH Osman SA Oreiby AF. Caseous lymphadenitis in sheep and goats: clinical, epidemiological and preventive studies. Small Rumin Res. (2009) 87:116–21. doi: 10.1016/J.SMALLRUMRES.2009.10.008

[16] Yitagesu E Alemnew E Olani A Asfaw T Demis C. Survival analysis of clinical cases of Caseous lymphadenitis of goats in north Shoa, Ethiopia. Vet Med Int. (2020) 2020:8822997. doi: 10.1155/2020/8822997 32879726 PMC7448112

[17] Son YS. Production and uses of Korean native black goat. Small Rumin Res. (1999) 34:303–8. doi: 10.1016/S0921-4488(99)00081-4

[18] Suh S Byun M Kim Y-S Kim M-J Choi S-B Ko Y-G. Analysis of genetic diversity and relationships of Korean native goat populations by microsatellite markers. J Life Sci. (2012) 22:1493–9. doi: 10.5352/JLS.2012.22.11.1493

[19] Kim KW Lee J Lee SS Kim S Lim HT Kim Y. Estimation of effective population size of Korean native black goat using genomic information. Int J Agric Biol. (2021) 25:575–80. doi: 10.17957/IJAB/15.1703

[20] Li H Yang H Zhou Z Li X Yi W Xu Y. Isolation, antibiotic resistance, virulence traits and phylogenetic analysis of *Corynebacterium pseudotuberculosis* from goats in southwestern China. Small Rumin Res. (2018) 168:69–75. doi: 10.1016/j.smallrumres.2018.09.015

[21] Aquino de Sá Mda C Gouveia GV Krewer Cda C Veschi JL de Mattos-Guaraldi AL da Costa MM. C and D genes in *Corynebacterium pseudotuberculosis* isolates from sheep and goats with caseus lymphadenitis. Genet Mol Biol. (2013) 36:265–8. doi: 10.1590/S1415-47572013005000013, PMID: 23885209 PMC3715293

[22] da Costa Barnabé NN Alves JRA de Farias AEM Alves FSF Faccioli-Martins PY Pinheiro RR. Assessment of caseous lymphadenitis in goats in a slaughterhouse in the Brazilian semi-arid region and estimates of economic losses due to carcass condemnation. Semin Ciências Agrárias. (2020) 41:2655–68. doi: 10.5433/1679-0359.2020v41n6p2655

[23] Tripathi BN Kumar J Sonawane GG Kumar R Dixit SK. Microbiological and molecular investigation of clinically suspected Caseous lymphadenitis cases in goats. Agric Res. (2016) 5:413–9. doi: 10.1007/s40003-016-0233-7

[24] Jung BY Lee SH Kim HY Byun JW Shin DH Kim D. Serology and clinical relevance of *Corynebacterium pseudotuberculosis* in native Korean goats (*Capra hircus* coreanae). Trop Anim Health Prod. (2015) 47:657–61. doi: 10.1007/s11250-015-0773-z, PMID: 25682104

[25] Kong J-Y Lee K Jung J-Y Kim JW Yoon N-S So B. Clinical case of internal caseous lymphadenitis in a native Korean goat (*Capra hircus* coreanae). J Prev Vet Med. (2019) 43:58–61. doi: 10.13041/jpvm.2019.43.2.58

[26] Olaifa AK. Comparison in haematological and biochemical changes in normal, acute and chronically castrated west African dwarf goats. Int J Res Med Sci. (2018) 6:1623. doi: 10.18203/2320-6012.IJRMS20181748

[27] Al-Gaabary MH Osman SA Ahmed MS Oreiby AF. Abattoir survey on caseous lymphadenitis in sheep and goats in Tanta, Egypt. Small Rumin Res. (2010) 94:117–24. doi: 10.1016/J.SMALLRUMRES.2010.07.011

[28] Tuncer SS Şireli HD Tatar AM. Behavioral patterns of goats. In: VII International Scientific Agriculture Symposium,” Agrosym 2016”. 6–9 October 2016. Jahorina, Bosnia and Herzegovina: Proceedings. University of East Sarajevo, Faculty of Agriculture. (2016). 2369–2374.

[29] Hefnawy A-E Youssef S Shousha S. Some Immunohormonal changes in experimentally pregnant toxemic goats. Vet Med Int. (2010) 2010:1–5. doi: 10.4061/2010/768438, PMID: 20613964 PMC2896860

[30] Thongkwow S Poosiripinyo N Pongkornkumpon N Saengsakchai S Klinkhiew N Chalatan T. Distribution and risk factors of clinical Caseous lymphadenitis in small-holder goat herds in northeastern Thailand. Thai J Vet Med. (2019) 49:343–51. doi: 10.56808/2985-1130.2999

[31] Kaba J Nowicki M Frymus T Nowicka D Witkowski L Szalus-Jordanow O. Evaluation of the risk factors influencing the spread of caseous lymphadenitis in goat herds. Pol J Vet Sci. (2011) 14:231–7. doi: 10.2478/v10181-011-0035-6, PMID: 21721407

[32] Sutherland SS Hart RA Buller NB. Genetic differences between nitrate-negative and nitrate-positive *C. pseudotuberculosis* strains using restriction fragment length polymorphisms. Vet Microbiol. (1996) 49:1–9. doi: 10.1016/0378-1135(95)00146-8, PMID: 8861638

[33] Ruiz JC D’Afonseca V Silva A Ali A Pinto AC Santos AR. Evidence for reductive genome evolution and lateral acquisition of virulence functions in two *Corynebacterium pseudotuberculosis* strains. PLoS One. (2011) 6:e18551. doi: 10.1371/journal.pone.0018551, PMID: 21533164 PMC3078919

[34] Soares SC Silva A Trost E Blom J Ramos R Carneiro A. The pan-genome of the animal pathogen *Corynebacterium pseudotuberculosis* reveals differences in genome plasticity between the biovar ovis and equi strains. PLoS One. (2013) 8:e53818. doi: 10.1371/journal.pone.0053818 23342011 PMC3544762

[35] Sellyei B Bányai K Bartha D Hajtós I Fodor L Makrai L. Multilocus sequencing of *Corynebacterium pseudotuberculosis* biotype Ovis strains. Biomed Res Int. (2017) 2017:1–7. doi: 10.1155/2017/1762162 PMC5660753 29159175

[36] Retamal P Ríos M Cheuquepán F Abalos P Pizarro-Lucero J Borie C. Host associated polymorphisms in the *Corynebacterium pseudotuberculosis* rpoB gene sequence. Vet Microbiol. (2011) 151:400–3. doi: 10.1016/j.vetmic.2011.03.012, PMID: 21482046

[37] Khamis A Raoult D La Scola B. rpoB gene sequencing for identification of Corynebacterium species. J Clin Microbiol. (2004) 42:3925–31. doi: 10.1128/JCM.42.9.3925-3931.2004, PMID: 15364970 PMC516356

[38] Guerrero J Oca-Jiménez R Dibarrat J León F Morales Erasto V Monroy SH. Isolation and molecular characterization of *Corynebacterium pseudotuberculosis* from sheep and goats in Mexico. Microb Pathog. (2018) 117:304–9. doi: 10.1016/j.micpath.2018.02.031, PMID: 29474828

